# Biosecurity on Cattle Farms: A Study in North-West England

**DOI:** 10.1371/journal.pone.0028139

**Published:** 2012-01-03

**Authors:** Marnie L. Brennan, Robert M. Christley

**Affiliations:** Department of Epidemiology and Population Health, Institute of Infection and Global Health, Faculty of Health and Life Sciences, Leahurst Campus, University of Liverpool, Liverpool, United Kingdom; Université de Toulouse, France

## Abstract

**Background:**

Few studies have considered in detail the range of biosecurity practices undertaken on cattle farms, particularly within the UK. In this study, 56 cattle farmers in a 100 km^2^ area of north-west England were questioned regarding their on-farm biosecurity practices, including those relating to animal movements, equipment sharing and companies and contractors visiting the farms.

**Methodology/Principal Findings:**

There was great variation between farms in terms of the type of, and extent to which, biosecurity was carried out. For example, the majority of farmers did not isolate stock bought onto the farm, but a small proportion always isolated stock. Many farmers administered treatments post-movement, primarily vaccinations and anthelmintics, but very few farms reported carrying out any health checks after moving animals on. In addition, there appeared to be much variation in the amount of biosecurity carried out by the different companies and contractors visiting the farms. Deadstock collectors and contracted animal waste spreaders, although likely to have a high potential for contact with infectious agents, were reported to infrequently disinfect themselves and their vehicles.

**Conclusions/Significance:**

These findings suggest that although certain biosecurity practices are undertaken, many are carried out infrequently or not at all. This may be due to many factors, including cost (in time and money), lack of proven efficacies of practices and lack of relevant education of veterinary surgeons, producers and other herd health specialists. Further research exploring the reasons for the lack of uptake is imperative if preventive medicine is to be utilised fully by the farming industry.

## Introduction

The term *biosecurity* has been defined in several ways. Frequently its scope is limited to ‘management systems that reduce the risk of introducing infectious disease to a herd’ [1] (i.e. external biosecurity). Management practices, including those affecting animal contacts within farms, may also affect disease spread between different animal management groups (i.e. internal biosecurity); despite the potential for impact on many diseases, in some cases this has been seen only as a backup system when between-farm systems have failed [2]. Although preventive techniques have been used for centuries to protect animals from disease [3], the term *biosecurity* came to the forefront of animal health in the UK during the foot and mouth disease (FMD) outbreak in 2001 [4], [5]. The continued association between FMD and biosecurity may affect the way many people interpret, and react to the term.

The putative benefits of undertaking biosecurity for disease prevention and/or control include improved production efficiency resulting in greater profits [6], [7], [8], [9], better animal welfare [10], improved immune responses to vaccines [3] and enhanced job satisfaction for producers, herd health professionals and other agricultural workers [11]. There exist recommendations for a wide range of biosecurity practices for the major livestock production systems, either for general disease prevention, or to minimise specific infection risks, including zoonotic risks. A number of studies have recommended biosecurity practices for cattle [2], [3], [12], [13], [14], [15], [16], [17], [18], [19], [20], [21], sheep [22], [23], [24], pig [25], [26], [27], poultry [28], alpaca [29] and fish [30] production systems. Whilst many of these studies advise the use of preventive procedures, they do not often provide evidence on the efficacies or cost-effectiveness of engaging in such practices. The few studies that do offer evidence of efficacy usually consider a single practice, such as disinfectant footbaths [31], [32], or look at the prevention of one disease only [33]. The considerable variation in recommendations between publications may lead to confusion amongst producers, resulting in them undertaking less appropriate practices. They may select practices that are ‘favoured’, or easy to implement, which may not be the most effective for that holding. [34].

Although some information exists for the UK [35], Sweden [36] and the USA [37], [38], there is generally little published data on the current use of preventive practices on cattle holdings. In order to optimise the use of preventive tools, it is important to understand first if and how they are being used. This can help to identify areas for further exploration, such as evidence-based research on the efficacy and cost-effectiveness of undertaking such practices. In addition, this knowledge is useful for investigation of other factors affecting producer decision-making related to biosecurity, such as sociological factors. This could assist producers and herd health advisors in deciding the most effective areas to invest in and could highlight areas requiring further producer/vet education and training. By investigating current behaviours ‘locally’, it is possible for regionally appropriate research to be undertaken or targeted education programs to be carried out, perhaps increasing the effectiveness of disease control and surveillance in an area.

The aim of this study, therefore, was to identify the biosecurity practices undertaken by cattle producers to prevent disease transmission within and between farms in a region.

## Materials and Methods

Cattle farmers within a 100 km^2^ area of north-west England were invited to participate in a cross-sectional study investigating contacts between cattle farms and any associated biosecurity practices undertaken. This study was part of a 3-tier research initiative which also investigated contacts between cattle herds on a national level [39] and a within-herd level [40].

All cattle farmers within this 100 km^2^ area were contacted via mail and given background information about the study. A follow up phone call, or visit to the farm if phone numbers were not available, determined whether farmers were willing to participate. As previously reported [41], from a total of 81 farmers that were approached, 56 farmers agreed to participate. Seven farms did not have cattle or were no longer trading livestock and 3 were shortly to cease trading. Thirteen farmers declined to participate, and 2 could not be contacted despite several attempts or could not make an appointment during the allotted data collection time. Therefore of the 71 farms currently trading cattle at normal capacity, 79% agreed to participate in the study and the results reported here relate to information elicited from these 56 farmers. Further details on the non-responders have been described previously [41]. The majority of farms visited were dairy farms (36 farms), with the remaining farms being fat-stock farms (19), suckler herds (15), store animal herds (8) and pedigree breeders (3) (farms could have more than one cattle enterprise). The median number of cattle per farm was 170 (Interquartile range (IQR) 104–320) and the median size of each farm was 80.3 hectares (IQR 48–137). The majority of farms (55) were family run businesses.

An interview-based questionnaire was designed to collect information from farmers or managers during visits to each of the 56 farms. This questionnaire can be found in Appendix S1. A pilot study involving 6 cattle farms outside of the study area was conducted prior to the commencement of the main study, and minor changes made to the questionnaire. The study data were collected between July and September 2005. The questionnaire was administered by the first author during face-to-face interviews and contained 191 questions; only those questions relevant to each individual farm were asked (e.g. if a farm exclusively ran a suckler enterprise, questions relating to visits by milk collectors were excluded). A selection of both closed and open questions were asked. Questions relating to contacts between the farms were included; these have been discussed in a previous publication [41]. Questions were also asked in relation to selected biosecurity practices; these practices were identified after review of the available literature. This information was gathered from peer-reviewed papers, government reports and advice sheets, and grey literature (non-conventional literature).

The practices selected related to activities surrounding animal movements, including the transport vehicles that were used and the isolation or treatment regimes undertaken after animals had moved onto a farm. Any biosecurity that was performed by producers relating to equipment sharing (the temporary lending and borrowing of equipment) between farms and any preventive measures undertaken by visiting company and contractor representatives were also examined. Additionally, behaviours surrounding waste disposal and permitting animal access to watercourses (streams, rivers etc.) were also explored. Practices relating to the reduction of within-farm transmission of pathogens were also investigated, particularly those related to housing, personnel and on-farm vehicles.

In this study, the term ‘imported’ refers to animals brought into the UK from another country. The term ‘shows’, ‘showed’ or ‘showing’ refers to animals taken to an agricultural event for judging. ‘Markets’ relate to animals being bought and sold at a livestock market. ‘Sales’ refers to animals being sold on a seasonal basis (e.g. bull sales) or as part of a cessation of trading event and can occur at various locations (e.g. market yards, farms). The expression ‘cleaning and disinfection’ in relation to personnel refers to individuals washing their outer protective clothing (including footwear) with water and/or disinfectant. The term ‘muck’ describes manure or faecal material from cattle, which in the UK is often collected and spread onto fields (‘muck spreading’).

Descriptive analyses were conducted in Microsoft Excel (Microsoft 2003). Univariable analyses were performed using Minitab Release 14.1 (Minitab Inc.) and SPSS 12.0.1 for Windows (SPSS Inc.). Fisher's Exact tests were used to investigate differences between the isolation of stock following introduction to the farm from different sources and whether access of stock to watercourses was affected by whether or not the watercourse traversed farmland upstream.

The study was conducted in accordance with the research ethics requirements of the Faculty of Veterinary Science at the University of Liverpool. Informed verbal consent was obtained from all participants involved in the study during initial phone calls or visits. Informed verbal consent was again obtained in person at the beginning of each interview and it was made clear to participants that by agreeing to be interviewed, they were agreeing to be part of the study.

## Results

### Preventing disease transmission between farms via direct contact

Risk associated with animal movements can be reduced by producers only purchasing animals from farms with a known disease history and through isolation, disease testing and prophylactic treatment of purchased stock. The proportion of farms reporting such measures in this study varied between the type of biosecurity practice and also by the origin of the animals pre-movement.

Of the 33 farmers that purchased stock directly from other farms, 70% (n = 23) reported that they inquired about the disease history of the vendor farm prior to purchase. Farmers indicated that the diseases of most concern were bovine viral diarrhoea (BVD; 57%, n = 13), bovine tuberculosis (bTB; 52%, n = 12), leptospirosis (43%, n = 10), infectious bovine rhinotracheitis (IBR; 26%, n = 6) and various respiratory conditions (17%, n = 4). Interestingly, 2 farmers (9%) nominated FMD, with only 1 farmer each nominating mastitis, *Salmonella* spp. and Johne's disease.

**Figure 1:**
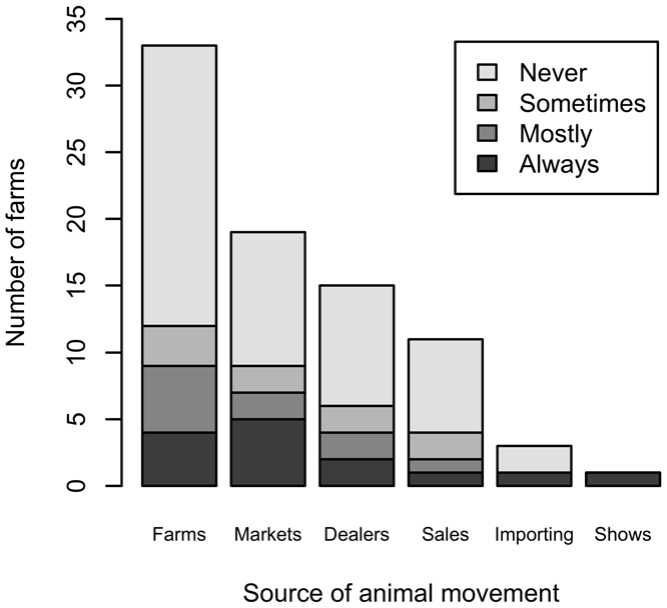
Number of farmers isolating stock after moving them onto the farm from various sources out of 56 farmers interviewed in a 100 km^2^ area of north-west England.

Most farmers did not isolate animals moved on from another farm, a market, a dealer or a sale (Figure 1). Few farms imported (n = 3) or showed (n = 1) animals. There were no significant associations between the various trading sources of animals and whether farmers ‘always’ or ‘never’ isolated stock on farm entry (Fishers Exact P-values 0.7–1).

**Figure 2:**
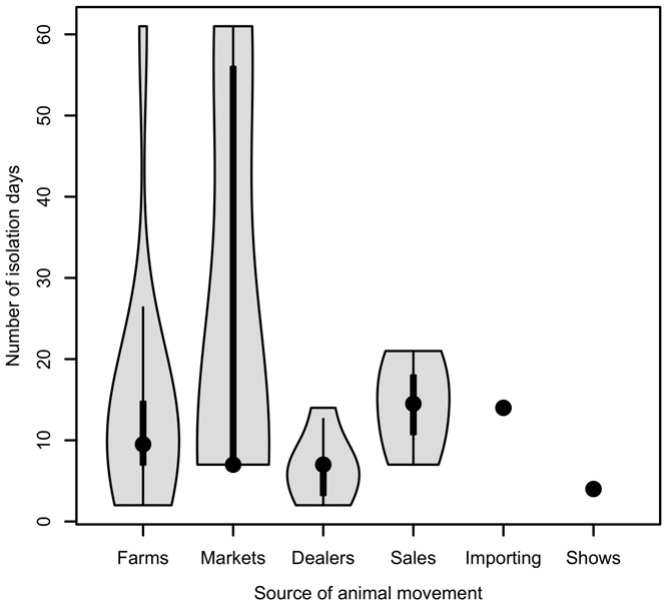
Violin plot of the number of days animals were isolated for after moving them onto the farm from various sources. Violin plots comprise a box-and-whiskers plot combined with a kernel density smooth to highlight the underlying frequency distribution of the data. Data are taken from interviews conducted with 56 farmers in a 100 km^2^ area of north-west England.

Farmers that imported animals and moved animals on from sales appeared to keep them in isolation for longer than those moving from other farms, markets, dealers and shows, although there was considerable variation (Figure 2). Six farms isolated stock moved on from some sources and not others. Re-analysis excluding these 6 farms indicated that animals from markets tended to be isolated for the longest period of time (median 32 days), followed by animals from sales (median 12 days), from other farms and dealers (median 7 days for both) and from shows (median 4 days).

**Figure 3:**
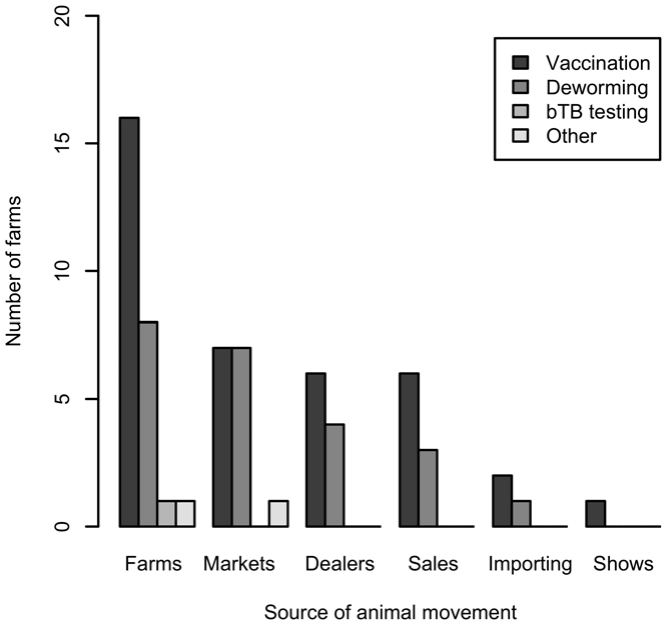
Number of farmers undertaking preventive measures on animals arriving onto the farm from various sources out of 56 farmers interviewed in a 100 km^2^ area of north-west England.

**Figure 4:**
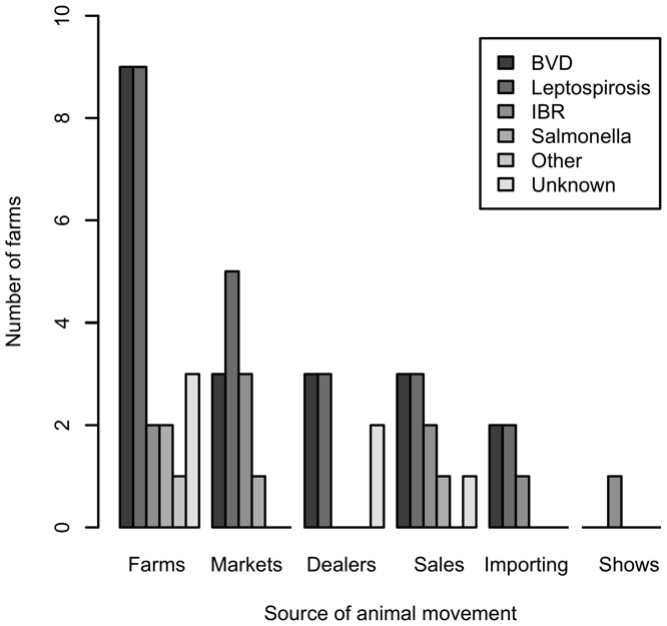
Number of farmers vaccinating animals against various diseases on their arrival onto the farm from various sources out of 56 farmers interviewed in a 100 km^2^ area of north-west England.

Treatment of stock post-movement was performed by more farms than were health checks or disease testing. The most common intervention was vaccination, followed by anthelmintic administration (Figure 3). The most common vaccines used were for protection against BVD and leptospirosis, followed by IBR and *Salmonella* spp. (Figure 4). Only 7 farms reported carrying out health checks after moving animals on; these related primarily to determining somatic cells counts in milk (n = 3) and blood tests for both BVDV and *Leptospira* (n = 3).

### Preventing disease transmission between farms via indirect contact (fomites: equipment, vehicles and personnel)

There was great variation between farms, and between companies and contractors in terms of whether vehicle and personnel biosecurity was carried out, or was seen to be carried out. Approximately 78% (n = 39) of the 50 producers trading through markets used their own vehicle for transporting animals, followed by 71% (n = 29) of the 41 producers trading with other farms, 39% (n = 11) of the 28 producers taking animals to slaughterhouses and 33% (n = 4) of the 12 producers buying or selling stock at sales. Only 7% (n = 2) of producers trading through dealers used their own vehicles to move animals for this purpose.

A list of the types of equipment shared between farms can be seen in Appendix S2. As previously reported in Brennan et al. [41], of the producers that shared equipment with other farmers (n = 24), 12 performed biosecurity on items before or after using them and 12 (50%) did not. Of the 5 farmers that lent items, 2 would clean items on their return, 2 would clean items prior to lending them and 1 farmer did both. Of the 8 farmers that borrowed items, 5 would clean the equipment prior to their return (1 farmer cleaned only one out of three items borrowed) and 2 would clean items prior to their use; 1 farmer did both. One farmer was included twice as they both lent and borrowed equipment (n = 13).

Overall, 28% (n = 215) of companies and contractors reportedly parked in animal areas (areas where animals had access to or were situated) and of these, most (89.8%; n = 193) never cleaned their vehicles after visiting farms (Table 1). As mentioned in Brennan et al. [41], those companies reported to be most likely to park in animal areas were hoof trimmers (94%, n = 17), muck spreaders (71%, n = 30) and deadstock collectors (47%, n = 26). The most likely companies to always clean vehicles after parking in animal areas were hoof trimmers (53%, n = 9), followed by muck spreaders (17%, n = 5) and milk companies (14%, n = 1). However, when focusing only on those organisations most likely to park vehicles in animal areas, deadstock collectors always cleaned their vehicles only 4% (n = 1) of the time.

**Table 1:** Biosecurity practices undertaken by companies and contractors as reported by interviewed farmers (n = 56) from a 100 km^2^ region of north-west England in 2005.

Companies/Contractors	Organisations parking in animal areas; % (No.)^1^	Cleaning of vehicles post visit; % (Frequency)^2^				Personnel going into animal areas; % (No.)^3^	Cleaning of personnel post visit; % (Frequency)^4^			
		Always	Sometimes	Never	Don't know		Always	Sometimes	Never	Don't know
AI technicians	8 (2/26)	0	0	100 (2/2)	0	100 (25/25*)	92 (23/25)	4 (1/25)	0	4 (1/25)
Animal hauliers	24 (7/29)	0	0	100 (7/7)	0	59 (17/29)	18 (3/17)	12 (2/17)	70 (12/17)	0
Bedding suppliers	31 (15/49)	7 (1/15)	0	93 (14/15)	0	2 (1/49)	100 (1/1)	0	0	0
Belly clippers	0 (0/1)					100 (1/1)	100 (1/1)	0	0	0
Castrators	50 (1/2)	0	0	100 (1/1)	0	100 (2/2)	0	0	50 (1/2)	50 (1/2)
Deadstock collectors	47 (26/55)	4 (1/26)	0	92 (24/26)	4 (1/26)	93 (51/55)	4 (2/51)	6 (3/51)	90 (46/51)	0
Drug company reps	0 (0/3)					0 (0/3)				
Farm assurance advisors	4 (2/46)	0	0	100 (2/2)	0	85 (39/46)	90 (35/39)	0	2 (1/39)	8 (3/39)
Feed/supplement suppliers	36 (19/53)	5 (1/19)	0	95 (18/19)	0	2 (1/53)	100 (1/1)	0	0	0
Fuel suppliers	20 (11/55)	0	0	91 (10/11)	9 (1/11)	0 (0/55)				
Government vets	14 (2/14)	0	0	100 (2/2)	0	100 (14/14)	100 (14/14)	0	0	0
Hedge trimmers	39 (15/38)	0	0	100 (15/15)	0	3 (1/38)	0	0	100 (1/1)	0
Hoof trimmers	94 (17/18)	53 (9/17)	0	47 (8/17)	0	100 (18/18)	83 (15/18)	0	6 (1/18)	11 (2/18)
Milk companies	19 (7/36)	14 (1/7)	0	86 (6/7)	0	0 (0/36)				
Muck spreaders	71 (30/42)	17 (5/30)	3 (1/30)	80 (24/30)	0	24 (10/42)	0	0	100 (10/10)	0
Other	24 (9/38)	11 (1/9)	0	89 (8/9)	0	26 (10/38)	80 (8/10)	0	10 (1/10)	10 (1/10)
Planters/harvesters	44 (14/32)	0	0	100 (14/14)	0	0 (0/32)				
Postman	4 (2/53)	0	0	100 (2/2)	0	0 (0/53)				
Private vets	7 (4/56)	0	0	100 (4/4)	0	100 (56/56)	96 (54/56)	4 (2/56)	0	0
Silage makers	42 (19/45)	0	0	100 (19/19)	0	0 (0/45)				
Tradespeople	14 (5/37)	0	0	100 (5/5)	0	0 (0/37)				
Trading Standards	0 (0/14)					36 (5/14)	80 (4/5)	0	20 (1/5)	0
Vermin controllers	36 (8/22)	0	0	100 (8/8)	0	50 (11/22)	18 (2/11)	0	64 (7/11)	18 (2/11)
**Total**	**28 (215/764)**	**8.8 (19/215)**	**0.5 (1/215)**	**89.8 (193/215)**	**0.9 (2/215)**	**34 (262/763** * **)**	**62 (163/262)**	**3 (8/262)**	**31 (81/262)**	**4 (10/262)**

1 Denotes number of farms where organisations park in animal areas/Total number of farms visited by that organization.

2 Denotes frequency of response/Number of farms where organisations park in animal areas.

3 Denotes number of farms where organisation personnel enter animal areas/Total number of farms visited by that organization.

4 Denotes frequency of response/Number of farms where organisation personnel enter animal areas.

*Denotes missing data from one farm.

Marginally more company and contractor personnel were reported to have contact with animals or animal areas (34%; n = 262) than vehicular contact with animal areas (28%; n = 215) (Table 1). However, the overall rate of company/contractor personnel reported to always clean and disinfect after coming into contact with animals or animal areas was 62% (n = 163), much greater than the value seen for the undertaking of vehicular biosecurity (8.8%, n = 19). Muck spreaders (n = 10) and hedge trimmers (n = 1) were reported to never undertake biosecurity (100%). Private veterinarians (100%; n = 56), deadstock collectors (93%; n = 51) and farm assurance advisors (85%; n = 39) had the largest number of personnel entering animal areas, as previously stated in Brennan et al. [41]. Private veterinarians were reported to always clean and disinfect themselves after visits 96% (n = 54) of the time, deadstock collectors 4% (n = 2) of the time and farm assurance advisors 90% (n = 35) of the time.

Only 36% of the 56 farms (n = 20) had regular routine veterinary visits occurring at a median of 26 times a year (IQR 13–26). Seventy three percent (n = 41) recorded herd health information about their animals, including any diagnoses made and any results from tests or surveys.

### Preventing disease transmission between farms via indirect contact (environment)

Risks associated with environmental transmission of pathogens can be reduced by preventing grazing of pastures recently spread with animal waste and preventing cattle access to common waterways. Almost all of the 56 farms (89%, n = 50) spread farm waste (manure, slurry, dirty water) onto land grazed by cattle. Most farmers waited a set interval before returning stock to grazing land spread with waste, with a median waiting time of 6 weeks (IQR 4–10). Few farms spread farm waste from other farms (4%, n = 2).

Most of the 56 farms had watercourses running through them (82%, n = 46); in the majority of cases these first crossed another farm prior to passing through each farmer's land (78%, n = 36). Cattle had access to waterways on approximately one-third (n = 13) of these 36 farms. Of the 22% of farmers (n = 10) nominating that the watercourse originated on their premises, 80% (n = 8) did not let cattle have access to the watercourse. The origin of a watercourse (on-farm or elsewhere) did not appear to be associated with a farmer's decision to allow cattle access to that watercourse (Fishers Exact P = 0.5).

### Preventing disease transmission within farms

Several questions were asked in relation to the prevention of disease transmission between different animals or animal groups within each farm. Of the 55 out of 56 farmers that housed animals, 75% (n = 41) always removed faecal material from pens before moving animals from different management groups into the pen. Just over half of the farmers (n = 31) responded that they routinely cleaned and/or disinfected housing after mucking out.

Tractors that were used for multiple tasks on 42 out of 56 farms (n = 50 tractors) appeared to be cleaned at varying frequencies. Some were cleaned between tasks (n = 2), some were cleaned a certain number of times per year (n = 19; median 4 times a year) and some were cleaned at varying frequencies (n = 29), including four that were infrequently, or never cleaned.

On only 7% of the 56 farms (n = 4) did farmers or their workers carry out any personal biosecurity (i.e. cleaning boots, changing overalls) between handling different management groups.

## Discussion

This study was one of the first to investigate the biosecurity measures undertaken by a sample of UK cattle farmers to reduce the risk of pathogen transmission within and between farms. We found considerable variation in how these measures were performed. This reflects the limited literature currently available that generally identifies that some farmers are undertaking little, or infrequent, biosecurity. A recent survey in the UK highlighted that 34% of sampled farmers stated that biosecurity was ‘almost non-existent’ on their farms [42]. This highlights the need for better understanding of factors underpinning farmers' decisions regarding implementation of biosecurity practices.

It has been suggested that there is a ‘lack of or inadequacy of public policy on biosecurity’ [43]. The current biosecurity recommendations for cattle farmers from DEFRA emphasize minimization of disease transmission between premises via contaminated clothing, vehicles and equipment [44], or specifically relate to FMD and other exotic diseases [45], [46] or bTB [47], [48] and provides little guidance on preventing transmission of endemic disease. The Scottish Agricultural College's website has more information relating to prevention of endemic diseases (http://www.sac.ac.uk/research/themes/animalhealth/animalhealthwelfare/biosecurity/) and goes as far as attempting to determine risk levels of specific activities (http://www.sac.ac.uk/research/themes/animalhealth/animalhealthwelfare/biosecurity/examples/). However, the available information regarding biosecurity may be of limited use to cattle producers due to a lack of clarity, inappropriate detail and lack of evidence of efficacy. Economic models have been constructed to examine the cost of animal disease [8], [49] but few or no intervention trials have been carried out to look at the cost-effectiveness of the recommended biosecurity practices.

Appropriate biosecurity is typically farm-specific and should be based on the diseases which have the greatest impact or those that the farm is at greatest risk of acquiring, where compliance is achievable and is within the economic capabilities of the producer [23], [50], [51]. Programs also need to be flexible in order to adapt to individual situations [50], [52]; this is important, as implementing biosecurity measures that are not suitable may lead to them becoming branded as ineffective or perceived as expensive and time inefficient.

The farms in our study appeared to be representative of lowland farming areas in the UK, with a typically higher average number of dairy animals per herd than the average across the UK [41]. For areas where other types of cattle enterprise predominate, it is possible that farmers would undertake practices differently, however common contacts such as animal movements and visits by companies and contractors and therefore associated risks are likely to occur. Basic enterprise information on the non-responding farmers appears to indicate that they are also typical of farmers in this area, although the effect on the data created by these non-responders is unknown.

### Between farm biosecurity – direct contacts

The majority of farmers in the current study inquired about the disease history of the vendor farm before purchasing stock. However, further information about what producers did to acquire and use such information was not collected; this should be investigated in future studies as relying on the appearance of an animal to indicate health status is risky [53]. In addition, the type of stock purchased may have an effect on whether vendor farm disease history is collected; this was not assessed in this study.

There is much information in the literature on diseases that can be purportedly acquired via purchasing cattle; Bazeley [54] contains an extensive list of these. Many farmers were concerned about stock contracting BVD and leptospirosis, two of the most common diseases in dairy herds in the UK [9]. Some producers were worried about their animals contracting bTB; at the time this study was conducted the north-west region of the UK was at relatively low risk for bTB compared to other areas such as the south-west of England and Wales (http://archive.defra.gov.uk/corporate/about/who/cvo/documents/2005report.pdf). We were initially surprised that two farmers nominated FMD as a particular concern as the 2001 FMD outbreak in the UK had been over for more than 4 years by the time of this study. However there is a growing body of evidence highlighting that this outbreak has had lasting social and psychological effects with members of the farming community experiencing substantial fear of another such disaster occurring [55]. These points highlight the human dimension of animal diseases and that thoughts and understanding of one disease or disease related issue (e.g. biosecurity) should not be viewed in isolation.

Quarantine of animals following arrival on a farm can be useful in reducing disease transmission between herds [56]. It is of concern that, despite recommendations from DEFRA, more than 50% of farmers in the current study did not isolate arriving stock regardless of their origin. This is similar to the results of a study of Irish farmers [57], but is much greater than reported for Swedish farmers [36]. Recommendations on isolation period length are somewhat unclear; DEFRA's information for livestock keepers on biosecurity only suggests that an isolation protocol be discussed with a private veterinarian [44]. A single published source recommends isolating animals for 21 days for diseases with short incubation periods [15]. In the current study there was considerable variation between the farms in terms of the duration of isolation, and reflects the variation found in a Swedish study [36]. In many cases, isolation length was also affected by the origin of the animals.

The low percentage of farms performing health checks on stock post-movement (9%) was the same as that seen in a previous study involving dairy farms (9%) [35], although the percentage giving routine treatments in the current study was much higher (between 60 and 70% for most movement types vs. 28% of farms). The reported use of vaccination reflects the diseases reported commonly by producers in this area (Brennan, unpublished data) and mirrors the vaccines used by dairy farms in a study conducted in Ireland [57].

### Between-farm biosecurity – indirect contacts

Equipment contaminated with mucus, faeces and blood can harbor infectious organisms and hence movement of equipment between farms may also move pathogens [19]. For this reason, it is recommended that borrowed or hired equipment be cleaned and disinfected [19], [44]. As identified in Brennan et al. [41], most farmers who borrowed equipment cleaned and disinfected the items prior to their return. This may suggest that the motivation for this may be socially driven, for example as an act of courtesy, rather than based on a perceived infection risk.

Transport vehicles can act as a transmission risk between farms [58], [59] and poor hygiene habits of companies and contractors visiting farms may result in the transmission of infectious diseases [60]. Non-farm vehicles should not be allowed on a farm unless essential [25] and if they are absolutely necessary, they should be clean and free of animal excreta [45]. The use of farm-owned vehicles for moving animals may reduce transmission risk and was commonly practiced in the study area. Transport by dealers may pose additional risks as they may make several pick-ups from multiple farms, potentially increasing the risk of infectious disease transmission.

This study found that many companies and contractors failed to undertake adequate biosecurity. As outlined in Brennan et al. [41], deadstock collectors are often considered a high biosecurity risk [37], [60] as they are likely to have contact with diseased animals; in this study they were nominated as cleaning and disinfecting vehicles infrequently. Similarly, muck spreaders visited more than half of the farms in the study area, yet reportedly cleaned and disinfected their vehicles infrequently. This is of particular concern considering the many diseases which can be transmitted via faeces [61], [62]. The lack of cleaning and disinfection of company and contractor vehicles was mirrored with evidence of little effort undertaken by company and contractor personnel to clean and disinfect themselves. The exception to this was private veterinarians, who reportedly cleaned and disinfected on the majority of farms. In a study by Noremark et al. [36], veterinarians were also reported to almost always use protective clothing on farms. Veterinarians should act as advisors regarding disease preventive practices as they are often nominated as the preferred source of information in relation to biosecurity [63], [64] and hence their behaviours may be highly influential.

Only 36% of farms had routine veterinary visits in this study. This is similar to a study of UK beef producers where approximately two-thirds of farmers had emergency only contact with their veterinary surgeon [65]. This may indicate that dialogue between many farmers and vets on general biosecurity and preventive practices may be unlikely to occur. General advice regarding preventive practices may be given during *ad hoc* visits but constraints on time and resources means this may not occur. The fact that the majority of farms are recording herd health information is encouraging; records are important for monitoring the success of herd health schemes, detecting emerging diseases and are an important baseline for the development of preventive programs [20], [21], [57].

In the UK, it is routine agricultural practice to remove manure and slurry from housing and place it in a secondary store for up to 3–6 months prior to spreading it on fields [66], [67] and such storage should destroy most bacteria, although certain pathogens such as *Cryptosporidium parvum* may require a longer time period due to their resistant nature [68]. Most farmers in the UK either continually remove slurry from sheds and place it in a secondary store, or remove waste from sheds between April and May (after animals have been moved outside). Spreading does not typically occur until Autumn-Winter (∼6 six months later) [66], therefore the risk of pathogen transmission from waste spread on fields is probably minimal, regardless of the length of time the fields have been left before being grazed.

Wildlife contact is mentioned in the literature as a potential source of pathogen transmission [13], [69]. Wildlife contact was not addressed here, or the measures producers undertook to prevent this contact.

### Within-farm biosecurity

There appears generally to be limited within-farm biosecurity carried out on farms in this area. This is likely to increase the risk for transmission of diseases among juvenile stock and older animals [15]. For example, young stock housed in pens that were scraped out and washed were half as likely to become infected with *C. parvum* as those that had only bedding removed [70].

Personnel moving between different management groups (including calves) within farms did not appear to undertake any cleaning protocols or change their attire between handling different management groups. In addition, tractors used for multiple tasks on the farms were cleaned at varying time intervals, sometimes infrequently. Therefore it is likely that the lack of within-farm biosecurity on these farms would increase the risk of transmission between different management groups. This may result in the perpetuation of disease within stock [37], and may create persistently infected adults by exposing young stock to pathogens.

This study relied on farmers reporting on the behaviours of visitors to the farm; as with most studies collecting information in this way, it is possible that these may not be representative of what visitors actually did.

### Conclusion

This study has identified that producers and farm visitors reportedly undertake biosecurity in a varied way, with some undertaking little or no preventive measures to combat disease transmission either within or between farms. Collecting baseline data such as these is an important first step to understanding why biosecurity is not undertaken more by individuals within the farming industry.

## Supporting Information

Appendix S1 (PDF)

Appendix S2 (DOCX)
