# Supplementary material for: Biosecurity on Cattle Farms: A Study in North-West England
Source: PLoS One. 2012 Jan 3;7(1):e28139. doi: 10.1371/journal.pone.0028139 (PMC3250388; doi:10.1371/journal.pone.0028139)
Supplement: Appendix S1 — (PDF) [file pone.0028139.s001.pdf]

43747

THE UNIVERSITY of LIVERPOOL

REGIONAL CONTACT NETWORK STUDY 2005

QUESTIONNAIRE

Marnie Brennan
Epidemiology Group
Department of Veterinary Clinical Sciences and Animal Husbandry
University of Liverpool
Leahurst, Neston
CH64 7TE

43747

BACKGROUND INFORMATION

Name:	
Address:	
Phone number:	
Mobile number:	
CPH number:	
OS (map) reference:	
Farm ID number:	

Aims of the study:

- To outline all direct and indirect contacts within and between farms in the study area
- To explore the concept of biosecurity and what types of measures are undertaken on farms currently
- To find out what people perceive as worthwhile biosecurity practices and who should be involved in the implementation and maintenance of such practices on farms

This can then be used to:

- Help us identify the role of particular farm management strategies and contact types in terms of the spread of infectious disease
- Provide the farming industry with feedback in relation to the current thinking on biosecurity and how it may be utilised more efficiently

This questionnaire will determine the types of animal movements and other forms of contact between farms. It will also determine the biosecurity practices currently undertaken by producers and their opinions on such practices.

This survey relates only to cattle unless otherwise specified. It also relates to any additional premises farmed.

All information collected in this survey is strictly confidential and will not be used or accessed by anyone else outside of this study. None of the data analysis will be linked personally to particular people or premises.

43747

1. What enterprises are there on the farm?

- [ ] Dairy
- [ ] Suckler beef
- [ ] Beef fattening
- [ ] Breeding sheep
- [ ] Sheep fattening
- [ ] Dealer
- [ ] Pedigree breeder
- [ ] Rearing stores
- [ ] Other ______

2. Size of farm: Acres ☐☐☐☐☐☐

Hectares ☐☐☐☐☐☐

CONTACTS WITHIN FARMS:

PART A: ANIMAL CONTACTS AND HOUSING

3. How many of the following animals are there on the farm?

Unweaned dairy calves	☐☐☐☐	Fattening bullocks	☐☐☐☐
Dairy heifers (less than 18m)	☐☐☐☐	Young bullocks less 18m	☐☐☐☐
Dairy heifers (18m - calving)	☐☐☐☐	Bull beef	☐☐☐☐
Fattening heifers	☐☐☐☐	Breeding bulls	☐☐☐☐
Lactating cows	☐☐☐☐	Dry cows	☐☐☐☐
Suckler calves	☐☐☐☐	Suckler cows	☐☐☐☐
Other	______	Store animals	☐☐☐☐

4. Do you routinely house any of these different animal groups in the same building at the same time (e.g. fattening bullocks with bull beef)?

- [ ] Yes
- [ ] No

5. Which of these groups normally share buildings?

______	Shared airspace? ☐	Nose-nose contact? ☐
______	Shared airspace? ☐	Nose-nose contact? ☐
______	Shared airspace? ☐	Nose-nose contact? ☐

43747

6. Do you always muck out pens before moving different groups of animals in? ☐ Yes ☐ No

7. If no, which groups do you not muck out between?

8. Do you routinely clean and/or disinfect pens after mucking out? ☐ Yes ☐ No

9. If yes, what do you use?

10. Do you run other livestock species owned by yourself on the farm? ☐ Yes ☐ No
If no got to Q14

11. If yes, which species and how many?

Sheep		Goats	
Pigs		Other	

12. Do you ever graze cattle with these animals together on the same pasture at the same time?
☐ Yes ☐ No

13. Which cattle groups would you graze them with?

43747

14. Do you ever have other livestock species not owned by yourself living on your farm?

☐ Yes ☐ No

15. If yes, what species and which farms do they originate from?

16. For what period of time are they normally on your farm?

43747

PART B: INDIRECT CONTACT THROUGH EQUIPMENT

17. Do any of the tractors used for waste management get used for other tasks?

☐ Yes ☐ No

18. What other tasks?

(i) ____ (iv) ____

(ii) ____ (v) ____

(iii) ____ (vi) ____

19. For those tractors you use for multiple tasks, how often do you clean them?

(i) ☐☐ ☐ Between tasks ☐ Per year ☐ Other ____

(ii) ☐☐ ☐ Between tasks ☐ Per year ☐ Other ____

(iii) ☐☐ ☐ Between tasks ☐ Per year ☐ Other ____

(iv) ☐☐ ☐ Between tasks ☐ Per year ☐ Other ____

(v) ☐☐ ☐ Between tasks ☐ Per year ☐ Other ____

(vi) ☐☐ ☐ Between tasks ☐ Per year ☐ Other ____

PART C: CONTACT VIA PERSONNEL

20. Between handling which groups of cattle (if any) would you or your employees:

☐ Dip boots ____

☐ Clean boots ____

☐ Change boots ____

☐ Clean clothes ____

☐ Change clothes ____

43747

CONTACTS BETWEEN FARMS:

PART A: ANIMAL MOVEMENTS

21. Do you run a closed herd? ☐ Yes ☐ No

22. When did you last move animals onto your farm? [ ]

The following questions relate to cattle movements

OTHER FARMS - ON

23. Have you bought cattle from other farms over the past 12 months? ☐ Yes ☐ No

24.Prior to purchasing animals from other farms do you find out about their disease history?

☐ Yes ☐ No ☐ Don't know

25. What diseases are you mostly concerned with?

[ ]

26. Which farms do you buy from regularly?

[ ]

27. How many of the movements on to your farm from other farms are for bull hiring? [ ][ ]

28. Which farms do you hire from regularly?

[ ]

43747

Isolation can be defined as the segregation of stock in an area that prevents any direct contact (i.e. nose-to-nose) with other stock

29. Do you isolate animals that have been purchased from another farm?

☐ Always ☐ Mostly ☐ Sometimes ☐ Rarely ☐ Never

30. How many days are they isolated for? ☐☐

31. Do you perform health checks or disease testing on newly purchased stock from other farms?

☐ Yes ☐ No

If yes, what do you do?

32. Do you give any sort of treatments to newly purchased stock from other farms?

☐ Yes ☐ No

If so, what?

OTHER FARMS - OFF

33. Have you sold cattle to other farms over the past 12 months? ☐ Yes ☐ No

34. If stock is sold to other farms, which farms do you sell to regularly?

35. How many of the movements to other farms are for bull hiring? ☐☐

36. Which farms do you hire out to on a regular basis?

43747

OTHER PIECES OF LAND:

37. Do you have additional farms or separate pieces of land that are under long term lease?

☐ Yes ☐ No

38. How many additional farms do you have cattle on? ☐☐

39. Which farms? (Number them)

40. Do you have additional farms or separate pieces of land that are under temporary lease? ☐ Yes ☐ No

41. How many of these do you have cattle on? ☐☐

42. Where is this land located? (Number them)

43. Do you have a sole occupancy authority (SOA)? ☐ Yes ☐ No ☐ Don't know

43747

OTHER PIECES OF LAND (CONT.) :

44.The following questions relate to any additional land that you might have

Premise Number	Record animal movements?			How many a year?	Separate personnel for additional farms?	
1.	A ☐	S ☐	N ☐	☐☐	☐ Yes	☐ No
2.	A ☐	S ☐	N ☐	☐☐	☐ Yes	☐ No
3.	A ☐	S ☐	N ☐	☐☐	☐ Yes	☐ No
4.	A ☐	S ☐	N ☐	☐☐	☐ Yes	☐ No
5.	A ☐	S ☐	N ☐	☐☐	☐ Yes	☐ No
6.	A ☐	S ☐	N ☐	☐☐	☐ Yes	☐ No
7.	A ☐	S ☐	N ☐	☐☐	☐ Yes	☐ No
8.	A ☐	S ☐	N ☐	☐☐	☐ Yes	☐ No
9.	A ☐	S ☐	N ☐	☐☐	☐ Yes	☐ No
10.	A ☐	S ☐	N ☐	☐☐	☐ Yes	☐ No
11.	A ☐	S ☐	N ☐	☐☐	☐ Yes	☐ No
12.	A ☐	S ☐	N ☐	☐☐	☐ Yes	☐ No
13.	A ☐	S ☐	N ☐	☐☐	☐ Yes	☐ No
14.	A ☐	S ☐	N ☐	☐☐	☐ Yes	☐ No

43747

OTHER PIECES OF LAND (CONT.) :

45. If no do people change/clean:

Premise Number	Their clothes and/or boots between the additional premise and the main premise?	If there is a difference between premises, what is the reason?
1.	Y ☐ N ☐ D ☐	
2.	Y ☐ N ☐ D ☐	
3.	Y ☐ N ☐ D ☐	
4.	Y ☐ N ☐ D ☐	
5.	Y ☐ N ☐ D ☐	
6.	Y ☐ N ☐ D ☐	
7.	Y ☐ N ☐ D ☐	
8.	Y ☐ N ☐ D ☐	
9.	Y ☐ N ☐ D ☐	
10.	Y ☐ N ☐ D ☐	
11.	Y ☐ N ☐ D ☐	
12.	Y ☐ N ☐ D ☐	
13.	Y ☐ N ☐ D ☐	
14.	Y ☐ N ☐ D ☐	

43747

OTHER PIECES OF LAND (CONT.) :

46. If yes, do they:

Premise Number	Tend stock on other farms not run by yourself?	If yes, do they undertake any biosecurity between the premises?
1.	☐ Yes ☐ No ☐ Don't know	☐ Yes ☐ No ☐ Don't know
2.	☐ Yes ☐ No ☐ Don't know	☐ Yes ☐ No ☐ Don't know
3.	☐ Yes ☐ No ☐ Don't know	☐ Yes ☐ No ☐ Don't know
4.	☐ Yes ☐ No ☐ Don't know	☐ Yes ☐ No ☐ Don't know
5.	☐ Yes ☐ No ☐ Don't know	☐ Yes ☐ No ☐ Don't know
6.	☐ Yes ☐ No ☐ Don't know	☐ Yes ☐ No ☐ Don't know
7.	☐ Yes ☐ No ☐ Don't know	☐ Yes ☐ No ☐ Don't know
8.	☐ Yes ☐ No ☐ Don't know	☐ Yes ☐ No ☐ Don't know
9.	☐ Yes ☐ No ☐ Don't know	☐ Yes ☐ No ☐ Don't know
10.	☐ Yes ☐ No ☐ Don't know	☐ Yes ☐ No ☐ Don't know
11.	☐ Yes ☐ No ☐ Don't know	☐ Yes ☐ No ☐ Don't know
12.	☐ Yes ☐ No ☐ Don't know	☐ Yes ☐ No ☐ Don't know
13.	☐ Yes ☐ No ☐ Don't know	☐ Yes ☐ No ☐ Don't know
14.	☐ Yes ☐ No ☐ Don't know	☐ Yes ☐ No ☐ Don't know

43747

MARKETS

47. Have you bought or sold cattle through markets over the past 12 months? ☐ Yes ☐ No

48. If stock is purchased from markets, which markets do you buy from regularly?

49. Do you isolate animals that have been moved onto your farm from a market?

☐ Always ☐ Mostly ☐ Sometimes ☐ Rarely ☐ Never

50. How many days are they isolated for? ☐☐

51. Do you perform any health checks or disease testing on newly arrived stock from markets?

☐ Yes ☐ No

If yes, what do you do?

52. Do you give any sort of treatments to newly arrived stock from markets?

☐ Yes ☐ No

If so, what?

53. If stock is sold through markets, which markets do you usually sell through?

54. Is your farm authorised for Multiple Pick Ups? ☐ Yes ☐ No ☐ Don't know

55. Is your farm authorised for Multiple Drop Offs? ☐ Yes ☐ No ☐ Don't know

43747

DEALERS

56. Have you bought or sold cattle through dealers over the past 12 months? ☐ Yes ☐ No

57. If stock is purchased from dealers, which dealers do you use mostly?

58. Do you isolate animals that have been moved onto your farm from a dealer?

☐ Always ☐ Mostly ☐ Sometimes ☐ Rarely ☐ Never

59. How many days are they isolated for? ☐☐

60. Do you perform any health checks or disease testing on newly arrived stock from a dealer?

☐ Yes ☐ No

If yes, what do you do?

61. Do you give any sort of treatments to newly arrived stock from a dealer?

☐ Yes ☐ No

If so, what?

62. If stock is sold through dealers, which dealers do you use mostly?

43747

SALES

63. Have you bought or sold cattle through sales over the past 12 months? ☐ Yes ☐ No

64. If stock is purchased from sales, which ones do you buy from regularly?

65. Do you isolate animals that have been moved onto your farm from a sale?

☐ Always ☐ Mostly ☐ Sometimes ☐ Rarely ☐ Never

66. How many days are they isolated for? ☐☐

67. Do you perform any health checks or disease testing on newly arrived stock from sales?

☐ Yes ☐ No

If yes, what do you do?

68. Do you give any sort of treatments to newly arrived stock from sales?

☐ Yes ☐ No

If so, what?

69. If stock is sold at sales, which ones do you sell through regularly?

SHOWS

70. Have you exhibited cattle at shows over the past 12 months? ☐ Yes ☐ No

71. If stock is exhibited at shows, which ones do you go to regularly?

72. Do you isolate animals that have been moved onto your farm from a show?

☐ Always ☐ Mostly ☐ Sometimes ☐ Rarely ☐ Never

73. How many days are they isolated for? ☐☐

74. Do you perform any health checks or disease testing on stock from shows?

☐ Yes ☐ No

If yes, what do you do?

75. Do you give any sort of treatments to stock from shows?

☐ Yes ☐ No

If so, what?

43747

BREEDING CENTRES

76. Have you taken cattle to a breeding centre over the past 12 months? ☐ Yes ☐ No

77. If you take stock to a breeding centre, which one do you usually go to?

78. Do you isolate animals that have been moved onto your farm from a breeding centre?

☐ Always ☐ Mostly ☐ Sometimes ☐ Rarely ☐ Never

79. How many days are they isolated for?

80. Do you perform any health checks or disease testing on stock from breeding centres?

☐ Yes ☐ No

If yes, what do you do?

81. Do you give any sort of treatments to newly arrived stock from breeding centres?

☐ Yes ☐ No

If so, what?

43747

SLAUGHTERHOUSES

82. Have you taken cattle directly to a slaughterhouse in the past 12 months? ☐ Yes ☐ No

83. If you take stock directly to a slaughterhouse, which one do you go to?

DEADSTOCK COLLECTORS

84. Do you normally:

- move the carcasses to the perimeter of the farm for them to collect?
  ☐ Yes ☐ No

- get them to pick the carcasses up from within the farm?
  ☐ Yes ☐ No

IMPORTING/EXPORTING

85. Have you in the past 12 months purchased or sold cattle to countries outside the UK?

☐ Yes ☐ No

86. If yes, which countries do you:

Sell to?

Buy from?

87. Do you isolate animals that have been imported?

☐ Always ☐ Mostly ☐ Sometimes ☐ Rarely ☐ Never

88. How many days are they isolated for?

89. Do you perform any health checks or disease testing on newly arrived stock that has been imported?

☐ Yes ☐ No

If yes, what do you do?

90. Do you give any sort of treatments to newly arrived stock that has been imported?

☐ Yes ☐ No

If so, what?

43747

OTHER

91. Do you do any other regular cattle movements on or off the farm? ☐ Yes ☐ No

92. If yes, specify:
The departure or destination point, whether you isolate animals moving on and for how long, whether you perform any health checks or treatments on newly arrived animals and how often the movements occur.

93. For which movements do you normally use your own vehicle?

- ☐ Market
- ☐ Show
- ☐ Sale
- ☐ Dealer
- ☐ Another farm
- ☐ Breeding centre
- ☐ Slaughterhouse
- ☐ Knackers yard
- ☐ None
- ☐ Other

94. Where do you normally clean and disinfect your vehicle after moving to/from:

A market	☐ At market	☐ At home
A sale	☐ At market	☐ At home
A slaughterhouse	☐ At slaughterhouse	☐ At home
A knackers yard	☐ At knackers yard	☐ At home

43747

PART B: DIRECT CONTACT BETWEEN ANIMALS BETWEEN FARMS

95. How many cattle escapes have there been into your farm from neighbouring farms over the past 12 months? ☐☐

96. Into which groups of cattle? ______

97. How many cattle escapes have there been out of your farm into neighbouring farms over the past 12 months? ☐☐

98. Which groups of cattle? ______

99. Do you isolate sick animals in your herd?

☐ Always ☐ Mostly ☐ Sometimes ☐ Rarely ☐ Never

100. If so, what percentage are isolated in the following locations?

Isolation area	☐☐☐		
Sick animal area	☐☐☐		
Calving box	☐☐☐		
Spare field	☐☐☐	Nose-nose contact with non-isolated animals?	☐
Spare pen	☐☐☐	Nose-nose contact with non-isolated animals?	☐
Other	☐☐☐	Nose-nose contact with non-isolated animals?	☐
Combinations of the above	______		

43747

PART C: INDIRECT CONTACT THROUGH EQUIPMENT FROM NEIGHBOURS

101. Below is a list of items that may be shared between farms. Which ones over the past 12 months have you shared with other farmers?

	Do you share?	Which farms?
Feed vehicles	☐	
Muck vehicles	☐	
Gates/crushes	☐	
Milking equipment	☐	
Calving equipment	☐	
Medicating equipment (e.g. drenching etc)	☐	
Tagging equipment	☐	
Clipping/hoof trimming equipment	☐	
Fencing equipment	☐	
Machinery for harvesting, ploughing	☐	
Cars/Motorbikes	☐	
Tractors/Trailers/ Wagons	☐	
Other 1	☐	
Other 2	☐	
Other 3	☐	

43747

102. Do you do any cleaning or disinfection of
[illegible]

	Yours		Theirs	
	When borrowed equipment is returned?	Before equipment is borrowed?	When you returned borrowed equipment?	Before you use borrowed equipment?
Feed vehicles	☐	☐	☐	☐
Muck vehicles	☐	☐	☐	☐
Gates/crushes	☐	☐	☐	☐
Milking equipment	☐	☐	☐	☐
Calving equipment	☐	☐	☐	☐
Medicating equipment (e.g. drenching etc)	☐	☐	☐	☐
Tagging equipment	☐	☐	☐	☐
Clipping/hoof trimming equipment	☐	☐	☐	☐
Fencing equipment	☐	☐	☐	☐
Machinery for harvesting, ploughing	☐	☐	☐	☐
Cars/Motorbikes	☐	☐	☐	☐
Tractors/Trailers/ Wagons	☐	☐	☐	☐
Other 1	☐	☐	☐	☐
Other 2	☐	☐	☐	☐
Other 3	☐	☐	☐	☐

43747

103. Have you in the past 12 months used colostrum from another farm?

☐ Yes ☐ No

104. If so, which farm?

105. Have you in the past 12 months used feed or bedding bought directly from another farm?

☐ Feed Which farm?

☐ Bedding Which farm?

43747

PART D: INDIRECT CONTACT VIA PERSONNEL BETWEEN NEIGHBOURS

106. Are any of your farm employees employed by other farmers (e.g. temporary milkers, farmhands)?
☐ Yes ☐ No

107. Do any of your employees run their own cattle farm? ☐ Yes ☐ No

108. Do you regularly socialise with any of your contiguous neighbours?
☐ Yes ☐ No

109. Which farms do they come from?

110. How often do you visit these neighbours?

☐ Daily ☐ Weekly ☐ Monthly ☐ Half yearly ☐ Yearly ☐ Less than yearly ☐ Never

111. How often do you get a visit from these neighbours?

☐ Daily ☐ Weekly ☐ Monthly ☐ Half yearly ☐ Yearly ☐ Less than yearly ☐ Never

112. Do you regularly socialise with people from other farms which are not contiguous?

☐ Yes ☐ No

113. Which farms do they come from?

114. How often do you visit these neighbours?

☐ Daily ☐ Weekly ☐ Monthly ☐ Half yearly ☐ Yearly ☐ Less than yearly ☐ Never

115. How often do you get a visit from these neighbours?

☐ Daily ☐ Weekly ☐ Monthly ☐ Half yearly ☐ Yearly ☐ Less than yearly ☐ Never

116. Do you go to local farmers meetings on a regular basis?

☐ Yes ☐ No

117. Please specify any other reason (apart from the ones mentioned already) why you would regularly visit contiguous or non-contiguous farms, which farms you visit, how often you visit them and how often they would visit you

43747

PART E: INDIRECT CONTACT VIA PERSONNEL AND EQUIPMENT (EXCLUDING CONTIGUOUS NEIGHBOURS)

118. The following questions relate to the various people or companies that may visit your farm

	Do you use them?		Name of company/person	How often?
Milk company	☐ Yes	☐ No		
Private vets	☐ Yes	☐ No		
Government vets	☐ Yes	☐ No		
Trading Standards/Local Authority	☐ Yes	☐ No		
AI technician	☐ Yes	☐ No		
Animal hauliers	☐ Yes	☐ No		
Deadstock collectors	☐ Yes	☐ No		
Feed/supplement suppliers	☐ Yes	☐ No		
Contractors: Muck spreaders	☐ Yes	☐ No		
Hoof trimmers	☐ Yes	☐ No		
Belly clippers	☐ Yes	☐ No		
Hedge trimmers	☐ Yes	☐ No		
Silage makers	☐ Yes	☐ No		
Planting/Harvesters	☐ Yes	☐ No		
Vermin control	☐ Yes	☐ No		
Castrators	☐ Yes	☐ No		
Farm assurance advisors	☐ Yes	☐ No		
Drug company reps	☐ Yes	☐ No		
Fuel suppliers	☐ Yes	☐ No		
Postman	☐ Yes	☐ No		
Tradespeople	☐ Yes	☐ No		
Bedding suppliers	☐ Yes	☐ No		
Other2	☐ Yes	☐ No		
Other3	☐ Yes	☐ No		

43747

	Do they park their vehicles in animal areas (areas where animals are situated or have access to)?	If yes, do they clean their vehicles after the visit?	If they personally come into contact with animals or animal areas, do they clean themselves after the visit?
Milk company	Y ☐ N ☐ D ☐	A ☐ S ☐ N ☐ D ☐	A ☐ S ☐ N ☐ D ☐
Private vets	Y ☐ N ☐ D ☐	A ☐ S ☐ N ☐ D ☐	A ☐ S ☐ N ☐ D ☐
Government vets	Y ☐ N ☐ D ☐	A ☐ S ☐ N ☐ D ☐	A ☐ S ☐ N ☐ D ☐
Trading Standards/Local Authority	Y ☐ N ☐ D ☐	A ☐ S ☐ N ☐ D ☐	A ☐ S ☐ N ☐ D ☐
AI technician	Y ☐ N ☐ D ☐	A ☐ S ☐ N ☐ D ☐	A ☐ S ☐ N ☐ D ☐
Animal hauliers	Y ☐ N ☐ D ☐	A ☐ S ☐ N ☐ D ☐	A ☐ S ☐ N ☐ D ☐
Deadstock collectors	Y ☐ N ☐ D ☐	A ☐ S ☐ N ☐ D ☐	A ☐ S ☐ N ☐ D ☐
Feed/supplement suppliers	Y ☐ N ☐ D ☐	A ☐ S ☐ N ☐ D ☐	A ☐ S ☐ N ☐ D ☐
Contractors:			
Muck spreaders	Y ☐ N ☐ D ☐	A ☐ S ☐ N ☐ D ☐	A ☐ S ☐ N ☐ D ☐
Hoof trimmers	Y ☐ N ☐ D ☐	A ☐ S ☐ N ☐ D ☐	A ☐ S ☐ N ☐ D ☐
Belly clippers	Y ☐ N ☐ D ☐	A ☐ S ☐ N ☐ D ☐	A ☐ S ☐ N ☐ D ☐
Hedge trimmers	Y ☐ N ☐ D ☐	A ☐ S ☐ N ☐ D ☐	A ☐ S ☐ N ☐ D ☐
Silage makers	Y ☐ N ☐ D ☐	A ☐ S ☐ N ☐ D ☐	A ☐ S ☐ N ☐ D ☐
Planting/Harvesters	Y ☐ N ☐ D ☐	A ☐ S ☐ N ☐ D ☐	A ☐ S ☐ N ☐ D ☐
Vermin control	Y ☐ N ☐ D ☐	A ☐ S ☐ N ☐ D ☐	A ☐ S ☐ N ☐ D ☐
Castrators	Y ☐ N ☐ D ☐	A ☐ S ☐ N ☐ D ☐	A ☐ S ☐ N ☐ D ☐
Farm assurance advisors	Y ☐ N ☐ D ☐	A ☐ S ☐ N ☐ D ☐	A ☐ S ☐ N ☐ D ☐
Drug company reps	Y ☐ N ☐ D ☐	A ☐ S ☐ N ☐ D ☐	A ☐ S ☐ N ☐ D ☐
Fuel suppliers	Y ☐ N ☐ D ☐	A ☐ S ☐ N ☐ D ☐	A ☐ S ☐ N ☐ D ☐
Postman	Y ☐ N ☐ D ☐	A ☐ S ☐ N ☐ D ☐	A ☐ S ☐ N ☐ D ☐
Tradespeople	Y ☐ N ☐ D ☐	A ☐ S ☐ N ☐ D ☐	A ☐ S ☐ N ☐ D ☐
Bedding suppliers	Y ☐ N ☐ D ☐	A ☐ S ☐ N ☐ D ☐	A ☐ S ☐ N ☐ D ☐
Other2	Y ☐ N ☐ D ☐	A ☐ S ☐ N ☐ D ☐	A ☐ S ☐ N ☐ D ☐
Other3	Y ☐ N ☐ D ☐	A ☐ S ☐ N ☐ D ☐	A ☐ S ☐ N ☐ D ☐

43747

119. Do you ever have people ☐ Fox hunting ☐ Game bird shooting on your land?

120. How often do they frequent the farm?

Huntsmen

Game bird shooters

The next few questions relate to the facilities that are available for cleaning and/or disinfection on your farm

121. Where are your facilities located?

(i)

(ii)

(iii)

(iv)

(v)

122. What do you have at each point?

Hose Bucket Brush Disinfectant Pump-pack sprayer
Carpet Pressure washer

(i)

(ii)

(iii)

(iv)

(v)

123. Are any of the facilities obvious so if I was a first time visitor to the farm I would know where at least one of the areas was?

☐ Yes ☐ No

43747

PART F: INDIRECT CONTACT VIA ENVIRONMENTAL FACTORS

124. The next section relates to the disposal of waste on your farm

	Do you spread it on land grazed by cattle?		If yes, at what point after application do you graze it?
Waste from your farm e.g. manure, slurry, dirty water	☐ Y	☐ N	☐ Appears clear<br>☐ After rain<br>☐ Set interval<br>☐ Other<br>☐ Don't know

Do you spread waste from other farms? If so, which farms?

Sewage from your farm	☐ Y	☐ N	☐ Appears clear<br>☐ After rain<br>☐ Set interval<br>☐ Other<br>☐ Don't know

Do you spread sewage from other farms/sources? If so, which farms/sources?

Abattoir waste	☐ Y	☐ N	☐ Appears clear<br>☐ After rain<br>☐ Set interval<br>☐ Other<br>☐ Don't know

Which abattoir ?

43747

125. Do you have any water courses (e.g. rivers, streams, burns etc) running through your farm?

☐ Yes ☐ No

126. Does it cross another farm before passing through your farm?

☐ Yes ☐ No

127. If so, which farm does it cross?

128. Do your cattle have access to it? ☐ Yes ☐ No

43747

PART G: OTHER AREAS OF POTENTIAL CONTACT

129. Do you run a B & B or any sort of farm stay? ☐ Yes ☐ No

130. Approximately how many visitors do you have staying with you per month? ☐☐

131. Can visitors come into contact with cattle or cattle areas?

☐ Yes ☐ No ☐ Don't know

132. Do you provide clothing or boots for them to wear? ☐ Yes ☐ No

133. If no, do you encourage them to use your facilities to clean their clothing or shoes?

☐ Yes ☐ No

134. Do you run a farm shop or sell any produce from your farm gate?

☐ Yes ☐ No

135. What do you sell?

136. Approximately how many people visit your farm to buy produce per month? ☐☐

137. Do people buying produce have any contact with cattle or cattle areas?

☐ Yes ☐ No

138. Do you run a demonstration farm? ☐ Yes ☐ No

139. Approximately how many visitors to the demonstration farm do you have per month?

☐☐

140. Do you have cattle as part of the demonstration farm? ☐ Yes ☐ No

141. Can people handle the cattle or have access to cattle areas? ☐ Yes ☐ No

142. Do you provide clothing or boots for people to wear whilst handling cattle or being in cattle areas?

☐ Yes ☐ No

143. If no, do you encourage people to use your facilities to clean or change their clothes or footwear after handling cattle or being in cattle areas?

☐ Yes ☐ No

43747

144. What sort of car parking surface do you have for visitors to the farm to park on?

- ☐ Hard-standing - bitumen, concrete
- ☐ Unsealed driveway
- ☐ Grass
- ☐ Loose gravel
- ☐ Other ____________

145. Do you routinely use the driveway to the dairy/sheds as a stock route?

☐ Yes ☐ No

146. If yes, how often?

☐ Daily ☐ Weekly ☐ Monthly ☐ Every 3 months ☐ Every 6 months ☐ Other ____________

147. Do you routinely use the driveway to the house as a stock route? ☐ Yes ☐ No

148. If yes, how often?

☐ Daily ☐ Weekly ☐ Monthly ☐ Every 3 months ☐ Every 6 months ☐ Other ____________

149. Do you routinely move cattle across public roads? ☐ Yes ☐ No

150. If yes, how often? ____________

151. Do you routinely move cattle along bridleways? ☐ Yes ☐ No

152. If yes, how often? ____________

43747

153. What percentage of time do you load animals from the following areas?

Designated area on perimeter of the farm ☐☐☐

Designated area within the farm ☐☐☐

From fields ☐☐☐

From housing ☐☐☐

154. What percentage of time do you unload animals into the following areas?

Designated area on perimeter of the farm ☐☐☐

Designated area within the farm ☐☐☐

Into fields ☐☐☐

Into housing ☐☐☐

155. How often do you have routine visits from your vet? ☐

156. Do you record herd health information about your animals including diagnoses made and results from any tests or surveys?

☐ Yes ☐ No

43747

BIOSECURITY

Biosecurity encompasses many things and different people have different ideas about what it actually means to be 'biosecure'. We are interested in discovering what people really think about biosecurity and what its perceptions are in the farming community.

157. What is your definition of biosecurity?

One definition of biosecurity is 'management systems that reduce the risk of introducing infectious disease to a herd'

158. What do you think about this definition?

159. Do you think this is similar to your own definition of biosecurity?

☐ Yes ☐ No

160. Please rate the following in terms of whether you think they are worthwhile biosecurity practices (see next page)

Very Useful   Useful   Not Very Useful   Don't know

43747

	Very useful	Useful	Not Very Useful	Don't know
Maintaining a closed herd	☐ VU	☐ U	☐ NVU	☐ DK
Buying animals from a farm of known disease status	☐ VU	☐ U	☐ NVU	☐ DK
Isolating animals moved onto a farm (including show animals)	☐ VU	☐ U	☐ NVU	☐ DK
Testing animals which have moved on	☐ VU	☐ U	☐ NVU	☐ DK
Using your own vehicle when transporting animals	☐ VU	☐ U	☐ NVU	☐ DK
Cleaning and disinfecting vehicles after moving animals	☐ VU	☐ U	☐ NVU	☐ DK
Isolating sick animals	☐ VU	☐ U	☐ NVU	☐ DK
Minimising contact between your animals and animals on neighbouring farms e.g. double-fencing	☐ VU	☐ U	☐ NVU	☐ DK
Not grazing different species together	☐ VU	☐ U	☐ NVU	☐ DK
Fencing off stock access to streams and watercourses	☐ VU	☐ U	☐ NVU	☐ DK
Not grazing animals on pastures that have been recently spread with waste (or resting pastures for an appropriate period of time before moving animals on)	☐ VU	☐ U	☐ NVU	☐ DK
Locating animal loading areas away from where animals are situated	☐ VU	☐ U	☐ NVU	☐ DK
Minimising the number of visitors to the farm by improving security (closing gates, seeing visitors by appointment only)	☐ VU	☐ U	☐ NVU	☐ DK
Ensuring visitors change or clean clothes and boots before and after coming into contact with stock or stock areas	☐ VU	☐ U	☐ NVU	☐ DK
Encouraging vehicles to park away from stock areas	☐ VU	☐ U	☐ NVU	☐ DK
Seeking regular advice from vets or herd health schemes on health issues	☐ VU	☐ U	☐ NVU	☐ DK
Regularly carrying out pest control	☐ VU	☐ U	☐ NVU	☐ DK
Minimising the sharing of equipment and machinery with other farms	☐ VU	☐ U	☐ NVU	☐ DK
Minimising the use of equipment and machinery for different purposes to avoid contamination e.g. avoiding feeding with vehicles used for muck handling	☐ VU	☐ U	☐ NVU	☐ DK

43747

43747

161. Which of these practices, if any, do you undertake on your farm?

Maintaining a closed herd	☐ Y	☐ N	☐ NA
Buying animals from a farm of known disease status	☐ Y	☐ N	☐ NA
Isolating animals moved onto a farm (including show animals)	☐ Y	☐ N	☐ NA
Testing animals which have moved on	☐ Y	☐ N	☐ NA
Using your own vehicle when transporting animals	☐ Y	☐ N	☐ NA
Cleansing and disinfecting vehicles after moving animals	☐ Y	☐ N	☐ NA
Isolating sick animals	☐ Y	☐ N	☐ NA
Minimising contact between your animals and animals on neighbouring farms e.g. double-fencing	☐ Y	☐ N	☐ NA
Not grazing different species together	☐ Y	☐ N	☐ NA
Fencing off stock access to streams and watercourses	☐ Y	☐ N	☐ NA
Not grazing animals on pastures that have been recently spread with waste (or resting pastures for an appropriate period of time before moving animals on)	☐ Y	☐ N	☐ NA
Locating animal loading areas away from where animals are situated	☐ Y	☐ N	☐ NA
Minimising the number of visitors to the farm by improving security (closing gates, seeing visitors by appointment only)	☐ Y	☐ N	☐ NA
Ensuring visitors change or clean clothes and boots before and after coming into contact with stock or stock areas	☐ Y	☐ N	☐ NA
Encouraging vehicles to park away from stock areas	☐ Y	☐ N	☐ NA
Seeking regular advice from vets or herd health schemes on health issues	☐ Y	☐ N	☐ NA
Regularly carrying out pest control	☐ Y	☐ N	☐ NA
Minimising the sharing of equipment and machinery with other farms	☐ Y	☐ N	☐ NA
Minimising the use of equipment and machinery for different purposes to avoid contamination e.g. avoiding feeding with vehicles used for muck handling	☐ Y	☐ N	☐ NA

43747

43747

162. If there is a difference between what you do on your farm and which practices you think are worthwhile, please give the main reason as to why this is the case

Maintaining a closed herd	
Buying animals from a farm of known disease status	
Isolating animals moved onto a farm (including show animals)	
Testing animals which have moved on	
Using your own vehicle when transporting animals	
Cleansing and disinfecting vehicles after moving animals	
Isolating sick animals	
Minimising contact between your animals and animals on neighbouring farms e.g. double-fencing	
Not grazing different species together	
Fencing off stock access to streams and watercourses	
Not grazing animals on pastures that have been recently spread with waste (or resting pastures for an appropriate period of time before moving animals on)	
Locating animal loading areas away from where animals are situated	
Minimising the number of visitors to the farm by improving security (closing gates, seeing visitors by appointment only)	
Ensuring visitors change or clean clothes and boots before and after coming into contact with stock or stock areas	
Encouraging vehicles to park away from stock areas	
Seeking regular advice from vets or herd health schemes on health issues	
Regularly carrying out pest control	
Minimising the sharing of equipment and machinery with other farms	
Minimising the use of equipment and machinery for different purposes to avoid contamination e.g. avoiding feeding with vehicles used for muck handling	

43747

163. Do you think that implementing a small number of biosecurity practices on your farm can be worthwhile or is it necessary to undertake all or most of them?

Need to do all ☐ Can do some ☐ Don't know ☐

164. If yes, which few do you think you could implement and still benefit from?

165. Do you think it is often more economic to treat disease on farm than to implement biosecurity measures?

☐ Yes ☐ No ☐ Don't know

166. Do you think it is often more time-efficient to treat disease on farm than to implement biosecurity measures?

☐ Yes ☐ No ☐ Don't know

167. Besides yourself, who else do you think should be involved in implementing and maintaining biosecurity on your farm?

43747

168. Over the past year which infectious diseases, if any, have you had significant problems with in your herd?

- ☐ Respiratory problems
  - ☐ IBR
  - ☐ Lungworm
  - ☐ Salmonella (calves)
  - ☐ Other
- ☐ Tuberculosis
- ☐ Abortions
  - ☐ BVD/MD
  - ☐ Leptospirosis
  - ☐ Campylobacter
  - ☐ Other
- ☐ Lameness
  - ☐ Digital dermatitis
  - ☐ Other
- ☐ Diarrhoea/Scours
  - ☐ Adults
    - ☐ Johnes disease
    - ☐ Salmonella
    - ☐ BVD/MD
    - ☐ Fluke
    - ☐ Campylobacter
    - ☐ Other
  - ☐ Calves
    - ☐ Cryptosporidium
    - ☐ Salmonella
    - ☐ Rotavirus
    - ☐ Coronavirus
    - ☐ Other
- ☐ Mastitis
  - Which organism?
- ☐ Skin infections
  - ☐ Udder
  - ☐ Ringworm
  - ☐ Other
- ☐ Other
- ☐ None

43747

169. What do you perceive the health status of your herd to be?

☐ Above average ☐ Average ☐ Below average ☐ Don't know

170. Where do you get information about biosecurity from?

171. Where would you prefer to get information about biosecurity?

☐ Research papers/journals
☐ Farming press
☐ Consultants/advisors
☐ Neighbours/other farmers
☐ Private vets
☐ Government vets
☐ Demonstration farms
☐ Television/Media
☐ Conferences
☐ Training courses
☐ Agricultural shows
☐ Salesmen/reps
☐ Farmers discussion groups
☐ The internet
☐ DEFRA
☐ Other

172. Whose advice would you be most likely to take about biosecurity issues?

☐ Research papers/journals
☐ Farming press
☐ Consultants/advisors
☐ Neighbours/other farmers
☐ Private vets
☐ Government vets
☐ Demonstration farms
☐ Television/Media
☐ Conferences
☐ Training courses
☐ Agricultural shows
☐ Salesmen/reps
☐ Farmers discussion groups
☐ The internet
☐ DEFRA
☐ Other

173. If you answered neighbours or other farmers, which farmers would you take advice from?

43747

174. Are there any other comments that you would like to make about biosecurity?

175. What is your view on the future of the farming industry in the UK?

- ☐ Very positive
- ☐ Positive
- ☐ Both positive and negative
- ☐ Negative
- ☐ Very negative
- ☐ Undecided

176. Is there anything else that we have discussed in this questionnaire that you would like to comment on?

Thank you very much for you time and your contribution to this study. Feedback from the questionnaire will be available after all the data has been collected and analysed.

43747

*177. Any additional information, comments for me to make

43747

*178. List all cattle movements over the last 12 months and the number of animals moved each time

	Date	No. of animals	On or Off		To/From location
1.			☐ On	☐ Off	
2.			☐ On	☐ Off	
3.			☐ On	☐ Off	
4.			☐ On	☐ Off	
5.			☐ On	☐ Off	
6.			☐ On	☐ Off	
7.			☐ On	☐ Off	
8.			☐ On	☐ Off	
9.			☐ On	☐ Off	
10,			☐ On	☐ Off	
11.			☐ On	☐ Off	
12.			☐ On	☐ Off	
13.			☐ On	☐ Off	
14.			☐ On	☐ Off	
15.			☐ On	☐ Off	
16.			☐ On	☐ Off	
17.			☐ On	☐ Off	
18.			☐ On	☐ Off	
19.			☐ On	☐ Off	
20.			☐ On	☐ Off	
21.			☐ On	☐ Off	
22.			☐ On	☐ Off	
23.			☐ On	☐ Off	
24.			☐ On	☐ Off	

43747

*178. (cont.)

	Date	No. of animals	On or Off		To/From location
25.			☐ On	☐ Off	
26.			☐ On	☐ Off	
27.			☐ On	☐ Off	
28.			☐ On	☐ Off	
29.			☐ On	☐ Off	
30.			☐ On	☐ Off	
31.			☐ On	☐ Off	
32.			☐ On	☐ Off	
33.			☐ On	☐ Off	
34,			☐ On	☐ Off	
35.			☐ On	☐ Off	
36.			☐ On	☐ Off	
37.			☐ On	☐ Off	
38.			☐ On	☐ Off	
39.			☐ On	☐ Off	
40.			☐ On	☐ Off	
41.			☐ On	☐ Off	
42.			☐ On	☐ Off	
43.			☐ On	☐ Off	
44.			☐ On	☐ Off	
45.			☐ On	☐ Off	
46.			☐ On	☐ Off	
47.			☐ On	☐ Off	
48.			☐ On	☐ Off	

43747

*178. (cont.)

	Date	No. of animals	On or Off	To/From location
49.			☐ On ☐ Off	
50.			☐ On ☐ Off	
51.			☐ On ☐ Off	
52.			☐ On ☐ Off	
53.			☐ On ☐ Off	
54.			☐ On ☐ Off	
55.			☐ On ☐ Off	
56.			☐ On ☐ Off	
57.			☐ On ☐ Off	
58,			☐ On ☐ Off	
59.			☐ On ☐ Off	
60.			☐ On ☐ Off	
61.			☐ On ☐ Off	
62.			☐ On ☐ Off	
63.			☐ On ☐ Off	
64.			☐ On ☐ Off	
65.			☐ On ☐ Off	
66.			☐ On ☐ Off	
67.			☐ On ☐ Off	
68.			☐ On ☐ Off	
69.			☐ On ☐ Off	
70.			☐ On ☐ Off	
71.			☐ On ☐ Off	
72.			☐ On ☐ Off	

43747

PART B: DIRECT CONTACT BETWEEN ANIMALS BETWEEN FARMS (CONT.)

179. Please indicate where the boundaries of your farms are (including any additional farms) on the maps provided

180. Please identify all bordering farms on the maps provided so that all your neighbours are identified

181. Please identify which of the perimeter fences that border your neighbours grazing land are non nose-to-nose contact fences e.g. double-fences, hedges etc. (but do not have roads, bridleways between them)

182. Number these fields on the map

183. Map out which management groups have gone into these fields over the last 12 months

Unweaned dairy calves	
Dairy heifers (less than 18m)	
Bullocks less than 18m	
Dairy heifers (18m-calving)	
Fattening bullocks	
Fattening heifers	
Bull beef	
Lactating cows	
Dry cows	
Breeding bulls	
Suckler cows	
Suckler calves	
Other (please specify)	

*184. View randomly selected perimeter fence that borders neighbours grazing land that is supposed to not allow nose-to-nose contact and grade it according to how much contact is possible.

Field number	Percentage contact	Other comments

43747

*185. How many main roads (i.e. public roads that people besides yourself drive on) do you have dividing/bordering your farm?

*186. How many public footpaths or bridleways do you have through/bordering your farm, including additional premises used for stock?

*187. What percentage of your land is bordered by woodland, including additional premises used for stock?

*188. How many different neighbours (including additional premises used for stock) do you have that are contiguous to your farm?

*189. Which neighbours?

*190. How many grazing fields do you have (including additional premises used for stock) that border your neighbours grazing land (that do not have roads, bridleways etc in between)?

*191. How many of your contiguous neighbours (including neighbours from additional premises used for stock) have grazing fields which border your grazing fields (not including fields that have roads, bridleways etc. between them)?
