# Supplementary material for: Biosecurity on Cattle Farms: A Study in North-West England
Source: PLoS One. 2012 Jan 3;7(1):e28139. doi: 10.1371/journal.pone.0028139 (PMC3250388; doi:10.1371/journal.pone.0028139)
Supplement: Appendix S2 — (DOCX) [file pone.0028139.s002.docx]

# Additional file

### Appendix 2- Types of equipment shared between the 56 farms within the study area

Calving equipment

Cars/Motorbikes

Feed vehicles

Fencing equipment

Gates/crushes

Machinery for harvesting/ploughing

Milking equipment

Muck vehicles

Other

Tagging equipment

Tractors/Trailers/Wagons
